# Thrombus composition and thrombolysis resistance in stroke

**DOI:** 10.1016/j.rpth.2023.100178

**Published:** 2023-05-16

**Authors:** Benoit Ho-Tin-Noé, Jean-Philippe Desilles, Mikael Mazighi

**Affiliations:** 1 Université Paris Cité, Inserm, Optimisation Thérapeutique en Neuropsychopharmacologie, Paris, France; 2 Interventional Neuroradiology Department and Biological Resources Center, Rothschild Foundation Hospital, Paris, France

**Keywords:** acute ischemic stroke, fibrinolysis, neutrophil extracellular traps, thrombolysis, thrombolysis resistance, thrombus composition

## Abstract

A State of the Art lecture titled “*Thrombus Composition and Thrombolysis Resistance in Stroke*” was presented at the ISTH Congress in 2022. Intravenous thrombolysis (IVT) remains the only pharmacologic option to re-establish cerebral perfusion at the acute phase of ischemic stroke. IVT is based on the administration of recombinant tissue plasminogen activator with the objective of dissolving fibrin, the major fibrillar protein component of thrombi. Almost 30 years on from its introduction, although the clinical benefits of IVT have been clearly demonstrated, IVT still suffers from a relatively low efficacy, with a rate of successful early recanalization below 50% overall. Analyses of thrombectomy-recovered acute ischemic stroke (AIS) thrombi have shown that apart from occlusion site, thrombus length, and collateral status, AIS thrombus structure and composition are also important modulators of IVT efficacy. In this article, after a brief presentation of IVT principle and current knowledge on IVT resistance, we review recent findings on how compaction and structural alterations of fibrin together with nonfibrin thrombus components such as neutrophil extracellular traps and von Willebrand factor interfere with IVT in AIS. We further discuss how these new insights could soon result in the development of original adjuvant therapies for improved IVT in AIS. Finally, we summarize relevant new data presented during the 2022 ISTH Congress.

## Essentials

• Thrombi causing the occlusion of a large intracranial artery are often resistant to tissue plasminogen activator (t-PA).
• Analyses of thrombi recovered by mechanical thrombectomy have helped understand t-PA resistance.
• Resistance to t-PA involves structural alterations of fibrin and nonfibrin fibrillar components.
• Pharmacologic targeting of nonfibrin thrombus components may help overcome tPA resistance.

## 1 Introduction

Before the introduction of intravenous thrombolysis (IVT) with recombinant tissue plasminogen activator (rt-PA) for acute ischemic stroke (AIS) in the 1990s [[1], [2], [3], [4], [5]] and its approval by the United States Food and Drug Administration in 1996, stroke management had been focused on poststroke secondary prevention and rehabilitation. By opening the way to emergency interventions at the acute phase of ischemic stroke, IVT has revolutionized stroke care. The rationale and objective of IVT are straight forward: to re-establish cerebral perfusion by dissolving the thrombus at the origin of the intracranial arterial occlusion and brain ischemia, via the administration of a drug targeting one of the thrombus cement elements, fibrin. Almost 30 years on from its introduction, although the benefits of IVT have been clearly demonstrated (ie, an increase in the proportion of patients with better neurologic and functional outcome) [1,6], IVT has suffered several significant limitations that have hindered its efficacy and broader use. These limitations include a narrow therapeutic window (within 4.5 hours after stroke onset) [7], a moderate rate of early recanalization efficacy (30%-40% overall and below 10% toward the most proximal occlusions) [4,8,9], and an associated increased risk of hemorrhagic complications [1,10].

The more recent development of mechanical thrombectomy (MT) as an alternative or adjunct recanalization therapy for AIS due to large vessel occlusion (LVO) has partly overcome the limitations of IVT. MT offers a widened therapeutic window (6 hours), achieves tremendous rates of successful recanalization (over 75%), and presents no inherent risk of hemorrhage [[11], [12], [13], [14], [15], [16], [17]]. Nonetheless, MT has limitations that prevent its widespread use and positioning as a universal and ultimate recanalization therapy for AIS. MT is indicated only for patients with AIS due to LVO in the anterior circulation (20%-30% of AIS) [[18], [19], [20]], and it requires access to specialized stroke centers with trained neurointerventionalists. Therefore, despite the progress brought by MT, there is still a significant potential and critical need for improvement of recanalization therapy, which remains the therapeutic cornerstone for improved functional outcomes and reduced mortality in AIS [21].

Interestingly, by enabling the collection of AIS thrombi, MT has provided the unique opportunity to study their composition, structure, and mechanical properties and to determine whether and how these parameters relate to each other and to various clinical variables, including etiology, time from onset, imaging, outcome, and response to recanalization therapies [[22], [23], [24], [25], [26], [27], [28], [29], [30], [31]]. With respect to the latter aspect of treatment response, recent analyses of MT-retrieved thrombi have provided new information on the mechanisms underlying IVT resistance in AIS, which could be instrumental in designing more effective IVT strategies that would benefit a wider patient population.

In this review, after a brief overview of the fundamental basis of IVT and notion of IVT resistance, we present recent advances in the understanding of the impact of AIS thrombus composition and structure on IVT efficacy and discuss promising therapeutic leads derived from these insights for improved IVT in AIS.

## 2 Basics of Intravenous Thrombolysis in Acute Ischemic Stroke

IVT is based on the administration of rt-PA, a serine protease whose proteolytic action allows the conversion of the single-chain proenzyme plasminogen into the active 2-chain plasmin, an enzyme with broad specificity but that is primarily responsible for fibrin degradation. Thrombolysis thus relies on promoting a natural process, fibrinolysis, with the intent of targeting a single but major component of the thrombus fibrillar network, fibrin.

**Figure 1:**
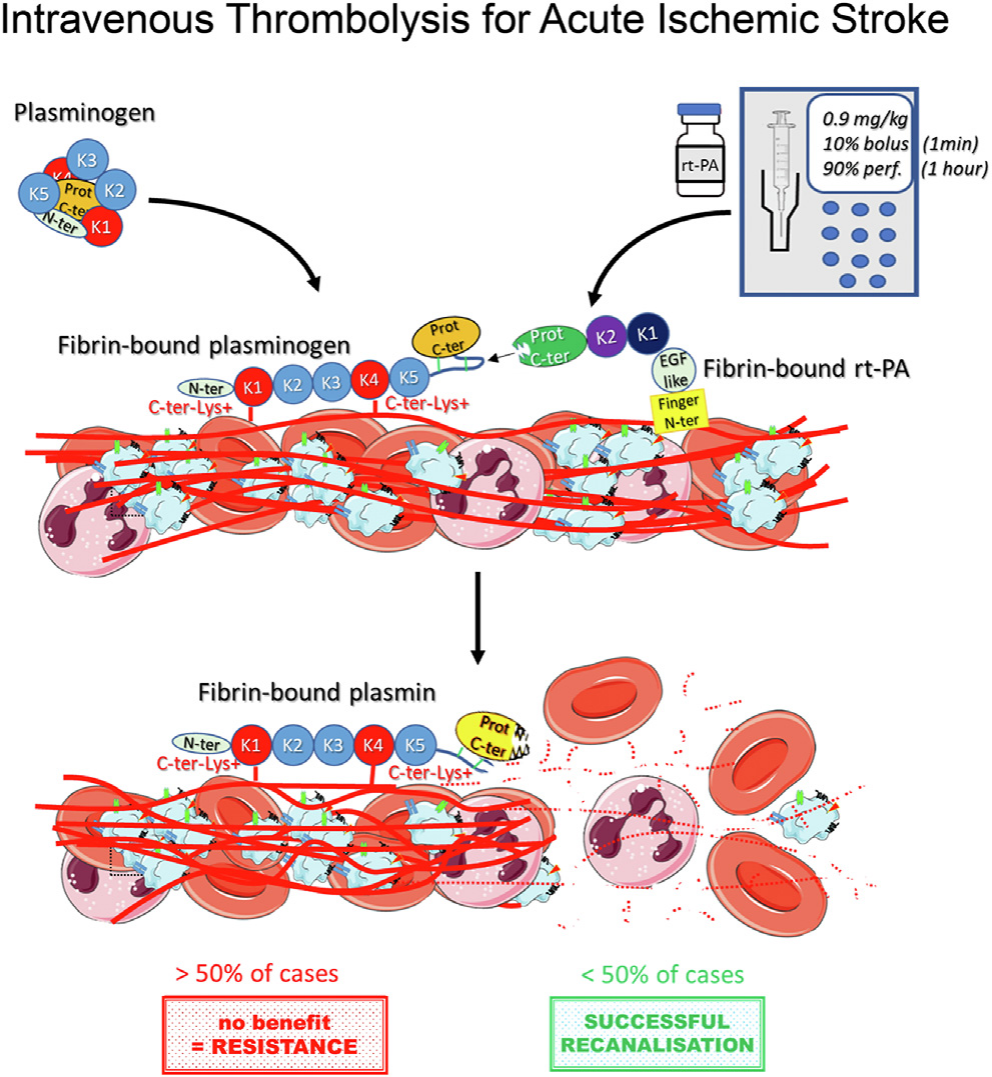
Schematic representation of the principle of intravenous thrombolysis for the treatment of acute ischemic stroke. C-ter, C-terminal; EGF-like, epidermal growth factor (EGF)-like domain; Lys, lysine; N-ter, N-terminal; perf, perfusion; Prot, protease domain; rt-PA, recombinant tissue plasminogen activator.

For optimal conversion of plasminogen into plasmin by t-PA, both plasminogen and t-PA must be bound to the fibrin surface (Figure 1). Fibrin indeed provides a template for the formation of a ternary activation complex between t-PA, plasminogen, and fibrin [[32], [33], [34]], before being subsequently degraded by plasmin. Assembly of this ternary activation complex is made possible by the fact that plasminogen and t-PA possess specific binding domains for fibrin: the plasminogen kringle 1 and 4 domains and the t-PA finger and kringle 2 domains. Binding of plasminogen and t-PA to fibrin not only concentrates them and brings them in proximity, but it also induces conformational changes in both plasminogen [[35], [36], [37]] and t-PA [38,39] that further promote their interaction (Figure 1). Fibrin thus acts as a cofactor for t-PA and improves considerably its catalytic efficiency (kcat/Km) toward plasminogen, which is much lesser in the presence of fibrinogen and poor in the fluid phase [33,34,40]. Because of these mechanistic features, at physiological blood concentration of plasminogen (1.5-2 μM), t-PA–mediated plasminogen activation occurs preferentially on fibrin surfaces. This reduces the risk of systemic plasminogen activation and uncontrolled proteolysis of fibrinogen and other circulating proteins by plasmin. Another mechanism that helps confine plasmin activity to the thrombus microenvironment is the instantaneous inhibition of free plasmin by the fast-reacting serpin α 2-antiplasmin, against which fibrin-bound plasmin is protected [4].

Importantly, the cofactor effect of fibrin toward plasminogen activation has long been known to be specific for t-PA and to not apply to other plasminogen activators such as urokinase and streptokinase [40]. The fibrin-selective character of t-PA has made of its recombinant form, rt-PA, the thrombolytic agent of choice for AIS. As compared with t-PA, urokinase and streptokinase display a less-favorable fibrinolytic/fibrinogenolytic activity ratio, and therefore an increased theoretical risk of systemic plasminogen activation, fibrinogenolysis, and α2-antiplasmin exhaustion [41,42], all of which undesired side effects can favor intracranial hemorrhage, the most devastating complication of AIS [43].

In AIS, rt-PA is classically administered as an initial 10% intravenous bolus over 1 minute followed by the continuous intravenous infusion of the remainder over 60 minutes for a total dose of 0.9 mg/kg (maximum dose, 90 mg; Figure 1) [44]. This high dose administration protocol is necessary to allow a continuous supply of active rt-PA to the thrombus for over an hour, because rt-PA is rapidly inactivated by endogenous plasminogen activator inhibitor 1 (PAI-1) and has a short initial plasma half-life (<6 minutes), and a terminal elimination half-life of 26 to 72 minutes [[45], [46], [47]]. Delays between bolus and infusion initiation may occur, which might affect rt-PA concentration and efficacy [44,48,49]. Tenecteplase, a genetically modified variant of rt-PA with increased fibrin specificity and plasma half-life (22 minutes), as well as a reduced susceptibility to PAI-1 [47], is emerging as a more convenient alternative to rt-PA. Because of its improved pharmacokinetics and activity compared with rt-PA, tenecteplase can be administered as a single bolus and does not require intravenous infusion [50]. Several regimens of tenecteplase have been tested, mainly 0.1, 0.25, and 0.4 mg/kg. Results from clinical trials have indicated that, besides its obvious practical advantages over rt-PA, tenecteplase at 0.25 mg/kg may be equally safe and as or more effective as rt-PA (0.9 mg/kg). Safety concerns have, however, been raised for the higher dose of 0.4 mg/kg [[51], [52], [53], [54], [55], [56], [57], [58], [59], [60]].

## 3 Resistance to Intravenous Thrombolysis in Acute Ischemic Stroke

Considering the natural specificity of rt-PA for fibrin, as well as its administration protocol that helps compensate for its short half-life and inhibition by circulating PAI-1, rt-PA appears as an ideal thrombolytic drug, which raises the question of why it fails in more than 50% of the cases. To address this question, it is important to define the concept of IVT failure or resistance in AIS. Surprisingly, to date, there is no clear definition of IVT resistance in AIS. For treating myocardial infarction (MI), IVT is considered a failure if it has not produced significant recanalization of the occluded artery (thrombolysis in myocardial infarction score ≥ 2, assessed by angiography) at 90 minutes after fibrinolytic administration (Table). Clinical evaluation criteria for successful thrombolysis in MI further include the resolution of clinical signs (ie, chest pain) and that of ST-segment elevation by more than 50%, within 60 to 90 minutes of fibrinolytic administration [[61], [62], [63]]. There are no well-established time, clinical, or recanalization score criteria for evaluating IVT success in AIS.

**Table:** The thrombolysis in myocardial infarction (TIMI) and revised thrombolysis in cerebral infarction (TICI) scales are intended to standardize the grading of *angiographic* assessment of perfusion following arterial recanalization therapy.

		TIMI flow scale
Grade 0		No perfusion = no antegrade flow beyond occlusion site
Grade 1		Penetration but no perfusion: the contrast material passes beyond the occlusion site but with minimal filling of the distal territory
Grade 2		Partial perfusion: the contrast material passes beyond the occlusion site and opacifies the distal coronary bed, incomplete or sluggish distal branch filling
Grade 3		Complete perfusion: full perfusion with antegrade filling of all distal branches, normal flow

		Revised TICI flow scale
Grade 0		No perfusion = no antegrade flow beyond occlusion site
Grade 1		Penetration but no perfusion: the contrast material passes beyond the occlusion site but with minimal filling of the distal territory
Grade 2	2a	Partial filling < 50% territory
	2b	Partial filling ≥ 50% territory
	2c	Near complete reperfusion except slow flow or few distal cortical emboli
Grade 3		Complete perfusion: full perfusion with filling of all distal branches, normal flow

In the treatment of myocardial infarction, thrombolysis is considered a success if the TIMI flow grade reaches 2 within 90 minutes of fibrinolytic administration. In the case of acute ischemic stroke, early recanalization has been defined as a TICI score ≥2b being reached within 3 hours of thrombolysis initiation.

The expression “Time is brain” was introduced in 1993 by Camilo R. Gomez to emphasize the rapidity of human nervous tissue loss as stroke progresses and stresses the urgent need for initiation of therapeutic interventions in AIS [[64], [65], [66]]. In line with this notion, for IVT to be considered successful in AIS, it must not only achieve arterial recanalization but must also do so rapidly. In 2 recent studies by Seners et al. [[67], [68], [69]], an early recanalization was defined as a thrombolysis in cerebral infarction score ≥2b being reached within 3 hours of IVT initiation (Table). Therefore, similar to that in MI, the notion of time and rapidity of action are central when assessing IVT success in AIS. With this in mind, it appears that clinical failure of IVT does not necessarily mean that rt-PA has failed to exert its biological action, ie, to promote the degradation of the fibrin network. Failed IVT may also occur if fibrinolysis is too slow or not sufficient in itself to cause early recanalization*.* The fact that imaging data have identified the larger and most proximal AIS thrombi, which are often the same, as those being the less responsive to IVT [8,67,69], supports the idea that IVT failure results from the inability to complete fibrinolysis in a timely manner. As described below, various endogenous fibrinolysis inhibition mechanisms likely contribute to the slowdown of fibrinolysis in AIS. Moreover, thrombolysis experiments in perfusion chambers have shown that thrombi can persist and even grow due to platelet accumulation during, and despite, effective fibrinolysis [[70], [71], [72], [73]]. The latter data stress how nonfibrin components can contribute to resistance to fibrinolysis-based thrombolysis by providing alternative thrombus frameworks. Analyses of MT-retrieved AIS thrombi have also shown that they contain nonfibrin scaffolding components that could lessen the efficacy of IVT.

Imaging studies have shown that better collaterals were independently associated with a higher rate of early recanalization and better functional outcome post-IVT [[74], [75], [76]]. The improved recanalization efficacy of IVT in individuals with good collaterals has been interpreted as a result of improved delivery of rt-PA to the thrombus [74]. Nonetheless, whereas rt-PA delivery issues may indeed represent a first obstacle to IVT efficacy, comparison of AIS thrombi composition and structure according to IVT status has indicated that rt-PA does reach AIS thrombi. In fact, results from several recent histologic and scanning electron microscopy studies are all converging to show that IVT induces changes in AIS thrombus structure. In particular, AIS thrombi from patients having received IVT prior to MT were slightly smaller, and displayed thinner and loosened fibrin fibers, compared with thrombi from patients treated directly by MT [[77], [78], [79], [80]].

In a recent study, we assessed the individual response of 16 AIS thrombi to *ex vivo* thrombolysis triggered by direct incubation of AIS thrombi with high doses of rt-PA and plasminogen. Our results showed that even in those conditions where delivery issues do not apply, the majority of AIS thrombi were fully or partially resistant to lysis, with rt-PA and plasminogen causing no or only partial reduction in thrombus weight within 1 hour of treatment [81]. In comparable experimental settings (ie, direct incubation of AIS thrombi in plasma supplemented with rt-PA), Laridan et al. [82] reported a mean decrease in AIS thrombus weight of approximately 40% after 2 hours of incubation with rt-PA. Very similar results were obtained in another study by Marder et al. [83] in which AIS thrombus area instead of thrombus weight was used as a readout for assessing thrombus lysis. Thus, it appears from these results that IVT resistance goes well beyond the sole matter of rt-PA delivery to the thrombus.

## 4 Components of Acute Ischemic Stroke Thrombi

In 2006, long before MT became the standard of care for AIS due to LVO, the first histologic analysis of human AIS thrombi was published by the group of Jeffrey Saver [84]. The observations reported in this seminal study still hold true: despite a marked variability in shape, size, as well as in the distribution of the thrombus cellular and fibrillar components, all AIS shared the same basic components. These components included red blood cells (RBCs), fibrin, platelets, and nucleated cells. Calcifications and cholesterol crystals were absent. Since then, the most notable new findings regarding human AIS thrombus composition have been the identification of von Willebrand factor (VWF) as a constitutive component of the thrombus fibrillar framework [[85], [86], [87]], and that of neutrophils as the main population of nucleated cells in AIS thrombi [81,82,[88], [89], [90]]. In addition, neutrophil extracellular traps (NETs) are now also recognized as substantial components of human AIS thrombi [25,81,82,[88], [89], [90], [91], [92], [93]]. Notably, the scarcity of calcifications and cholesterol crystals in AIS thrombi was recently confirmed in a large retrospective multicenter study [94]. Although some recent studies have suggested that AIS thrombus composition is dynamic, changes associated with thrombus aging have been shown to affect the respective proportions but not the nature of the various thrombus components [89,95]. Taken together, these data show that, as varied as they are, AIS thrombi have a common framework made of platelets, RBCs, fibrin, VWF, neutrophils, and NETs, with the distribution and proportions of these components representing their major distinguishing factors.

## 5 The Structural Organization of Acute Ischemic Stroke Thrombi

**Figure 2:**
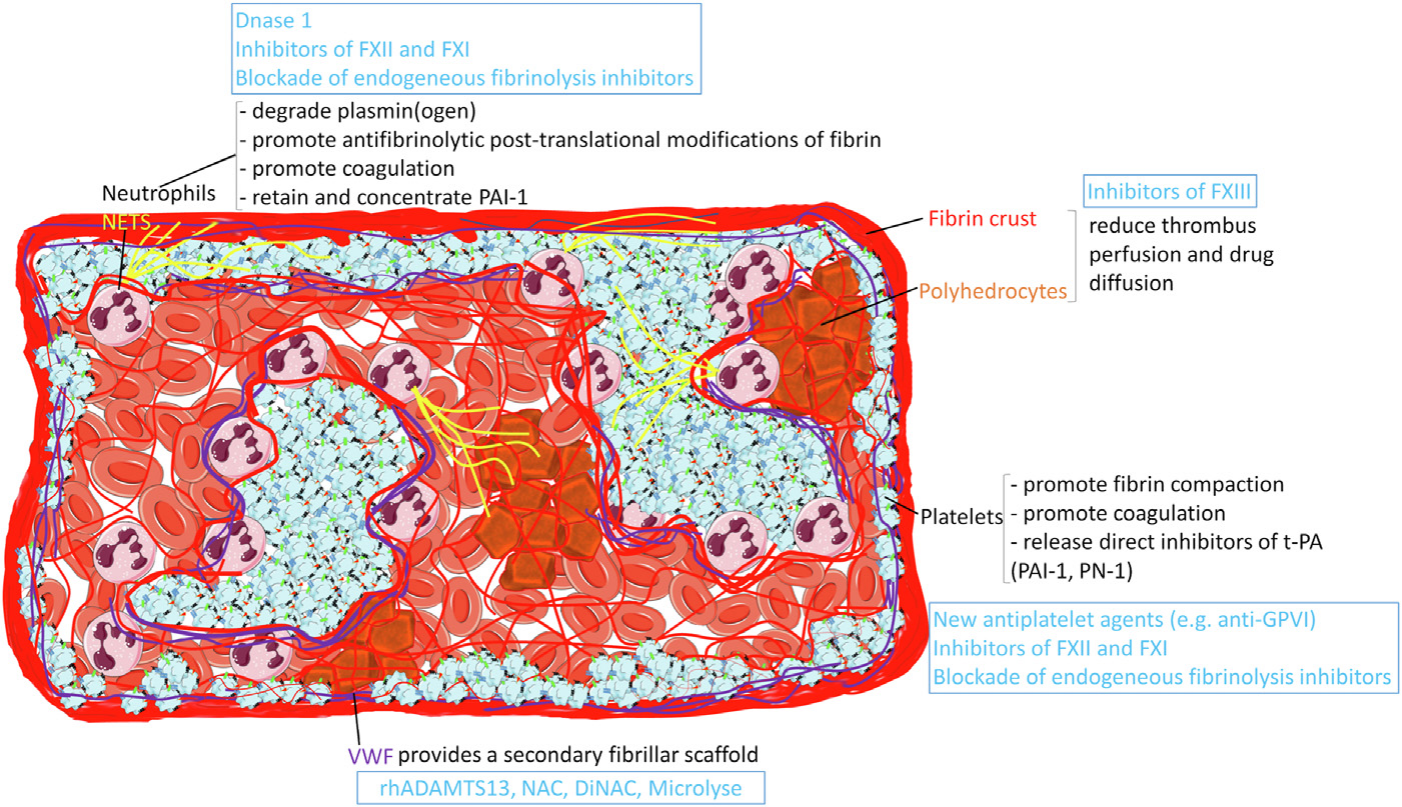
The mechanisms of fibrinolysis resistance in acute ischemic stroke thrombi and the pharmacologic options to antagonize them. F, factor; GPVI, glycoprotein VI; NAC, *N*-acetylcysteine; DiNAC, *N,N'*-diacetyl-L-cystine; PAI-1, plasminogen activator inhibitor 1; PN-1, protease nexin 1; t-PA, tissue plasminogen activator; VWF, von Willebrand factor.

Another common feature of human AIS thrombi pertains to their structural organization. There are converging data from several groups indicating that AIS thrombi comprise 2 major types of regions with different susceptibilities to rt-PA–mediated thrombolysis: RBC-rich areas and platelet-rich or composite areas [25,77,[86], [87], [88],^92^,^96^]. RBC-rich areas contain an abundance of RBCs trapped in a mesh of sparse thin fibrin fibers [86,87,93,96], with some RBCs displaying the shape of polyhedrocytes [77,87,93], which was previously shown to be indicative of clot retraction and compaction [[97], [98], [99]]. Platelet-rich or composite areas are mainly composed of platelets and contain leukocytes, mostly neutrophils but no or few RBCs [77,86,87,93,96]. Their fibrillar network contains VWF and NETs, alongside densely matted and compressed fibrin [77,86,87,93,96]. The inner core of AIS thrombi can vary from a homogeneous RBC-rich area to the other extreme of an almost exclusively platelet-rich area with little RBCs. Yet, in the majority of cases, the inner core is heterogeneous and presents a mixture of juxtaposed RBC- and platelet-rich areas [86]. The outer layer of AIS thrombi consists mostly of a platelet-rich area of various thicknesses, covered by a crust of dense fibrin [87,93,96,100], whose hermetic aspect [87,93,96] closely resembles that of the fibrin biofilm covering hemostatic blood clots [101]. The formation and assembly of the platelet-rich peripheral shell of AIS thrombi likely involve several nonexclusive mechanisms. First, we have shown that platelets were crucial for its formation [87]. The presence of platelets at the periphery of AIS thrombi could result from clot contraction, which has been shown to cause a redistribution of platelets to the periphery [97,102]. It could also be the mere reflection of platelet margination in the arterial circulation [103]. Fibrin compression might be due to the combined mechanical action of contracting platelets and hemodynamic pressure [[104], [105], [106]]. Besides mechanical forces, several posttranslational modifications alter fibrin morphology and organization. These modifications include factor XIII–mediated transglutamination [107,108], ferric iron-induced oxidation [109], nitrotyrosination [108,110], carbamylation [111], and even exposure to platelet factor 4 [112]. Concerning IVT resistance, the peripheral compaction and densification of AIS thrombus components are likely to impair the perfusion and diffusion of rt-PA to the thrombus core [113]. Furthermore, posttranslational modifications of fibrin such as carbamylation have been shown to reduce the cofactor effect of fibrin toward t-PA–mediated plasminogen activation [111]. In accordance with these data, we have shown that, as suggested by their histologic features [86], the shell and platelet-rich regions of AIS thrombi display a decreased susceptibility to rt-PA, as compared with RBC-rich regions [87]. This indicates that the components of these regions contribute to IVT resistance and may, therefore, represent targets for improving the thrombolytic efficacy of rt-PA (Figure 2) [24,25,92,93].

## 6 Platelets and Thrombolysis Resistance

As the name implies, platelets are central components of platelet-rich regions. Platelets not only participate in clot compaction but also contain and release the 2 major direct inhibitors of t-PA, PAI-1 and protease nexin 1 [114], which were found in abundance in platelet-rich, rt-PA–resistant regions of AIS thrombi [87]. Although PAI-1 is a short-lived inhibitor of rt-PA in the circulation, recent results have shown that a functional pool of PAI-1 remains anchored to the platelet membrane following platelet activation, promoting local fibrinolysis resistance [115]. Platelets may also interfere with fibrinolysis via promotion of coagulation (Figure 2). Procoagulant platelets are highly activated platelets that stimulate thrombin generation by expressing phosphatidylserine and P-selectin at their surface [116]. Coagulation was previously shown to interfere directly with rt-PA–mediated thrombolysis in mice [117]. Interference of coagulation with IVT is also illustrated by the fact that in the treatment of MI, the best recanalization efficacy of thrombolysis was found when combining fibrinolytics, including rt-PA, with anticoagulation [62,118,119].

## 7 Von Willebrand Factor and Thrombolysis Resistance

There are contradictory data regarding the impact of VWF on t-PA-mediated fibrinolysis, with reports of either impaired or enhanced fibrinolysis in the presence of VWF [120,121]. Yet, irrespective of a possible modulatory effect toward fibrinolysis and fibrin structure [121], VWF contributes to IVT resistance by providing a secondary fibrillar support frame to AIS thrombi that may help to compensate partly for the loss of fibrin. Recent results indeed indicate that VWF in AIS thrombi is not affected by rt-PA treatment [91]. Interestingly, several experimental studies in mice have shown that drugs targeting VWF exert a thrombolytic effect toward rt-PA–resistant thrombi (Figure 2) [86,122,[123], [124], [125]].

## 8 Neutrophils and Thrombolysis Resistance

There is strong and converging evidence that the antithrombolytic effect of neutrophils depends on the release of NETs. In 2010, Fuchs et al. [126] showed that NETs could provide a fibrinolysis-resistant scaffold sufficient to support clot formation despite rt-PA–mediated fibrin degradation. Mangold et al. [127] later showed that DNase 1 could accelerate rt-PA–mediated lysis of human coronary thrombi, which contained NETs. Remarkably, the results by Mangold et al. [127] brought the proof of concept that DNase 1 could be used not only as an antithrombotic but also as an adjuvant for thrombolytic therapy. Simon De Meyer’s and our groups have found that NETs are constitutive components of AIS thrombi, and confirmed that the potential of DNase 1 as an adjuvant to rt-PA for thrombolytic therapy also applies to these thrombi [82,90]. Notably, whereas drugs targeting VWF have standalone thrombolytic activity in mice [85,122,[123], [124], [125]], DNase 1 has no major lytic effect on human AIS thrombi in the absence of rt-PA [90]. This suggests that, in contrast to fibrin and VWF, extracellular DNA plays only a minor role in the structural stability of AIS thrombi. We have recently shown that the thrombolytic benefits brought about by degradation of extracellular DNA in human AIS thrombi come from enhancement of rt-PA–mediated fibrinolysis [81], thus indicating that NETs in AIS thrombi are antifibrinolytic. Several antifibrinolytic activities of NETs have been described. Direct inhibition of fibrinolysis by NETs can occur via degradation of plasminogen by NETs-associated neutrophil elastase [128] and via retention of PAI-1 in the thrombus [91]. As for platelets, another mechanism by which NETs may interfere with fibrinolysis is through the promotion of coagulation. Neutrophils and NET components have well-known procoagulant properties due to the ability of extracellular DNA to ignite the contact phase by providing a negatively charged surface, and to that of neutrophil elastase to degrade tissue factor pathway inhibitor and thrombomodulin (Figure 2) [129,130].

## 9 Potential Determinants of Thrombus Composition and Structure

Considering the importance of thrombus composition and structure in IVT resistance, identifying factors that influence these parameters may help better predict response to IVT. The determinants are most likely multifactorial, encompassing stroke etiology (eg, cardioembolic vs large artery atherosclerosis) [131,132] and underlying conditions (eg, cancer) [133], risk factors, comorbidities, and inflammation, all of which factors can be interrelated. With respect to etiology, there is converging evidence that AIS thrombi from cardioembolic origin have higher leukocyte, DNA, and NET content as compared with AIS thrombi linked to large artery atherosclerosis [82,131,134,135]. Whether the higher DNA and NET content of AIS thrombi from cardioembolic origin translates into higher resistance to IVT remains to be determined. Several risk factors for ischemic stroke are known to affect fibrin structure and susceptibility to thrombolysis. For instance, diabetes has been shown to induce the formation of denser, less-porous clots, with increased resistance to fibrinolysis [[136], [137], [138]]. Likewise, a higher concentration of fibrinogen also leads to increased fibrin density and resistance to fibrinolysis [139,140], and data from patients with MI have shown an independent link between plasma clot permeability and resistance to fibrinolysis and intracoronary thrombus fibrin content [141]. Recently, lipoprotein(a), which possesses antifibrinolytic properties by competing with plasminogen for binding to fibrin [142,143], was confirmed to be a risk factor for AIS [144]. Infections and inflammation may also impact AIS thrombus composition and susceptibility to thrombolysis. Analyses of blood clots and thrombi from COVID-19 patients have indicated that COVID-19 was associated with compact fibrin, increased resistance to fibrinolysis, and possibly higher NET content, including in AIS thrombi [82,[145], [146], [147]].

## 10 Toward New Adjuvant Therapies for Improved Intravenous Thrombolysis in Acute Ischemic Stroke

As discussed above, DNase 1 has largely proven its efficacy as an adjuvant to rt-PA in *ex vivo* thrombolysis assays using human AIS thrombi [81,82,90]. Moreover, it was previously shown to be well tolerated when administered intravenously to patients with lupus [148]. Its clinical formulation, Dornase alfa (Pulmozyme), has entered 2 phase 2 trials (EXTEND-IA DNase, NCT05203224 and NETS-target, and NCT04785066) aiming at evaluating its efficacy in patients who had an AIS treated with IVT and eligible for MT. Notably, however, because Pulmozyme is initially intended for inhalation, its current formulation and conditioning are not optimal for intravenous administration and limit the dosage amount that can be administered to patients who had an AIS.

In contrast to DNase 1, drugs targeting VWF present the originality of having proved their thrombolytic potential irrespective of rt-PA use, at least in mice [85,122,124,125]. Moreover, these drugs are already in clinical use, which could ease their repurposing toward a new indication such as AIS. These drugs include *N*-acetylcysteine (NAC, the active ingredient of HIDONAC) [122] and recombinant human disintegrin and metalloprotease with thrombospondin type 1 repeats 13 (rhADAMTS-13, the recombinant form of the endogenous protease responsible for the degradation of VWF multimers) [85,125]. Interestingly, recent data have suggested that the disulfide dimer of NAC, *N,N'*-diacetyl-L-cystine, may have an increased thrombolytic potential toward rt-PA–resistant thrombi, as compared with NAC [124]. To date, although the thrombolytic action of NAC, *N,N'*-diacetyl-L-cystine, and rhADAMTS-13 has been shown in various experimental models of thrombosis [85,122,124,125], their effect on AIS thrombus degradation in *ex vivo* thrombolysis assays remains to be assessed.

Conventional anticoagulation with heparin or direct oral anticoagulants on top of rt-PA cannot be envisioned in AIS due to the obvious risk of precipitating hemorrhagic transformation [149]. New drugs targeting factors XI and XII could provide adjuvant anticoagulant therapies well suited for AIS treatment because of their increased safety profile. Moreover, these selective inhibitors of the coagulation contact pathway appear particularly relevant for targeting NET-dependent coagulation [150].

Antiplatelet drugs also present an inherent risk of hemorrhagic complications that has limited their use in AIS [151]. The development of new antiplatelet agents targeting noncanonical pathways of platelet activation has opened the way for a reconsideration of antiplatelet strategies in AIS. Interestingly, after successfully completing a phase 1b/2a trial (ACTIMIS study, NCT03803007), glenzocimab, a humanized Fab fragment to platelet glycoprotein VI that blocks collagen-dependent platelet activation without compromising hemostasis [152,153], has entered an adaptive phase 2/3 study for AIS (ACTISAVE study, NCT05070260).

## 11 ISTH Congress Report

The 2022 ISTH Congress was an opportunity to learn new insights on the mechanisms of fibrinolysis resistance and to discover new therapeutic leads for improving thrombolytic therapies.

Ollivier et al. [154] showed that carbamylation occurred in AIS thrombi in relation to the presence of neutrophils. Consistent with predictions from *in vitro* studies, fibrin carbamylation in AIS thrombi had a negative impact on the response to both IVT and MT, by increasing resistance to fibrinolysis and thrombus stiffness.

Kadaba Ramanujam et al. [155] showed that fibrinogen concentration was a critical determinant of fibrin structure and clot resistance to rupture, as increasing fibrinogen concentration resulted in fewer, thicker fibrin fibers, and increased clot mechanical toughness. Gauer et al. [156] reported that the interaction between fibrin and platelet glycoprotein VI stimulated the development of platelet procoagulant activity, thereby increasing fibrin fiber density and reducing plot porosity.

By combining a mathematical and experimental approach, Risman et al. [114] strengthened the notion that densification of the fibrin network at the clot periphery acts as a temporary barrier for t-PA diffusion into the clot, thus impairing external fibrinolysis of contracted clots.

Solonomenjanahary et al. [157] provided quantitative data showing that IVT was associated with a significant increase in t-PA content of AIS thrombi, thus supporting the notion that IVT resistance does not result from rt-PA delivery issues. Instead, they showed that the thrombolytic action of IVT-derived rt-PA could be triggered by degrading extracellular DNA with DNAse 1, thus identifying NETs in AIS thrombi as being responsible for blocking the activity of intravenously-administered rt-PA.

A presentation by Marc van Moorsel was dedicated to a new thrombolytic agent, microlyse, a fusion protein consisting of a high-affinity single variable domain on a heavy chain antibody (VHH or nanobody) to VWF combined with the protease domain of urokinase-type plasminogen activator [158]. Interestingly, the superiority of microlyze over rt-PA in reducing ischemic injury following FeCl3-induced cerebral thrombosis in mice was shown.

Finally, Mathews et al. [159] showed that CPI, a natural carboxypeptidase inhibitor, accelerated rt-PA–mediated thrombolysis in mice, when given prior to thrombolytic therapy initiation.

## 12 Future Directions

The analysis of AIS thrombus composition in the last years has already led to the identification of several promising targets for the improvement of IVT. Future directions in the field of IVT in the setting of AIS should focus on translating these findings into clinical practice. Several drugs with known safety profiles are readily available to target the nonfibrin AIS thrombus components of interest. This should facilitate the design of prospective randomized trials, as illustrated by the start of the EXTEND-IA DNase and NETS-target trials in 2022.

## 13 Conclusion

The analysis of AIS thrombus composition has been a fast-growing field of research since the development of MT as an alternative or complementary therapy to IVT for achieving early recanalization in patients with occlusion of proximal large vessels. In addition to its tremendous recanalization efficacy, MT may thus prove instrumental in helping design the future of IVT, which still holds a notable potential, either as a standalone or bridging therapy, as access to MT remains limited in many places.
